# Perspectives of healthcare providers on withdrawal of life-sustaining treatment and advanced directives for unresponsive wakefulness syndrome in China

**DOI:** 10.3389/fneur.2024.1358747

**Published:** 2024-08-14

**Authors:** Meiqi Li, Siyu Dai, Le Wang, Haibo Di

**Affiliations:** ^1^International Unresponsive Wakefulness Syndrome and Consciousness Science Institute, Hangzhou Normal University, Hangzhou, China; ^2^Intensive Care Unite, Hangzhou First People’s Hospital, Hangzhou, China; ^3^School of Clinical Medicine, Hangzhou Normal University, Hangzhou, China; ^4^School of Basic Medicine, Hangzhou Normal University, Hangzhou, China

**Keywords:** advanced directives, disorders of consciousness, unresponsive wakefulness syndrome, withdrawal of life-sustaining treatment, ethics

## Abstract

**Objectives:**

We performed the current research to describe healthcare providers’ perspectives toward withdrawal of life-sustaining treatment (WLST) and advanced directive (AD) of patients with unresponsive wakefulness syndrome (UWS) and to identify influencing factors of their perspectives.

**Methods:**

Healthcare providers were recruited during a professional conference on disorders of consciousness (DoC). Participants completed self-administered questionnaires which included demographics, personal perspectives regarding WLST and the perception of ADs.

**Results:**

A total of 230 Chinese healthcare providers (female: 69.7%) were included. Only a small proportion reported positive attitudes toward withdrawing artificial nutrition and hydration (35.2%), antibiotics (30.9%), and do-not-resuscitation orders (23.5%) in UWS patients. As for predictors’ identification, religion was significantly associated with the positive attitude toward DNR order (*p* = 0.004). Moreover, although 47.4% of the participants had never heard of ADs before of conference, almost all of them would consider ADs (95.7%) thereafter, especially for non-neurologists (*p* = 0.033).

**Conclusion:**

The propensity to WLST for UWS in China is low and perspective on WLST is significantly associated with individual characteristics. The attitudes of healthcare providers toward integrating ADs in the decisional process are positive. Future research regarding ADs and their predictors should be carried out to improve the quality of end-of-life care of UWS in China.

## Introduction

Following severe brain injury, damage to neural pathways associated with arousal and awareness can lead to disorders of consciousness (DoC). The progression to vegetative state (VS)/unresponsive wakefulness syndrome (UWS) begins with the recovery of wakefulness without any indication of awareness (1, 2). Only about 20% of UWS patients can recover consciousness, which is a notably low percentage, and unfortunately, these prognoses are largely due to the misdiagnosis of patients with locked-in syndrome or cognitive-motor dissociation as UWS (3). They can become disabled and bedridden, suffering from permanent motor, cognitive, and speech impairments (4).

Life-sustaining treatment (LST) supports or replaces vital organ function by using mechanical ventilation, renal dialysis, chemotherapy, antibiotics, and artificial nutrition and hydration. However, the underlying condition of the disease cannot be altered by using LST. In recent decades, the ethical debate about LST has been very heated (5–7). Because of consciousness and speech impairments, UWS patients can hardly express their will. As a result, there is a risk that persistent LST may violate the ethical principle of the patient’s best interest and autonomy (8). Studies on the attitudes of healthcare providers toward end-of-life care were carried out in several western countries such as Italy, Britain, and Germany (9–13). A European survey showed that 80% of patients’ families believed that the condition of UWS was worse than death (9). More than two-thirds of the physicians believed that withdrawal of life-sustaining treatment (WLST) in UWS was appropriate (9). In China, cultural tradition complicates the implementation of WLST as discussing death is generally disapproved of as disrespectful (14, 15). Previous research demonstrated that Chinese people frequently prioritize families’ opinions over the autonomy of patients when making medical decisions (5, 16). Hence, the WLST and ADs have been rarely studied in the Chinese context. Notably, Shenzhen became the first city in China to achieve legislation on ADs in June 2022 (17). According to this law, healthy individuals are allowed to express their medical preferences verbally, in writing, or by other means when conscious, and hospitals and families are required to follow the patient’s ADs when making decisions. The impacts of this recent AD legislation on public’s perception regarding medical decision-making remains unknown, particularly for the perspective of healthcare professionals (18).

With the huge population of China, the absolute number of patients with brain injury may exceed that of most countries, causing a huge financial and mental burden to society and families (19, 20). Given the cultural taboos, end-of-life services for the public are a neglected area of political endeavor. And it is unclear what is the Chinese perspective on WLST and AD. In this light, this current study aimed to investigate healthcare providers’ perceptions of WLST and ADs in patients with UWS and to identify the factors which may influence the medical decision-making process.

## Methods

### Ethical approval

This study was approved by the Ethics Committee of Hangzhou Normal University (Ref No. 2022050). Informed consents were obtained from the participating healthcare providers. Completion of the questionnaire was anonymous. The study was conducted strictly according to the World Medical Association’s Declaration of Helsinki.

### Participants

Participants were recruited during a professional conference on DoC hosted in September 2022, organized by the International Unresponsive Wakefulness Syndrome and Consciousness Science Institute in China. Before circulating the questionnaire, an experienced neuroscientist provided a detailed overview of the definition, classification, diagnosis, treatment, and rehabilitation of DoC to the recruited healthcare providers. The ethical dimension of end-of-life care for DoC was also addressed (see Supplementary material). WLST was defined as “the deliberate discontinuation of life-sustaining treatment, without providing an alternative, with the understanding that this will lead to the patient’s death” (11). AD was defined as “a tool for exercising autonomy, the aim is to enable surrogates to make decisions on behalf of the person in the best possible manner in line with the values and interests of the person (21). And AD often is a statement about situations/conditions where a person, e.g., would not want to receive life-sustaining treatment, but rather only would want to receive palliation and be allowed to die.” A common understanding of the concept of the UWS was introduced to attendance in the conference before distributed the questionnaire: A severe brain injury in which the survivor is unable to perceive their surroundings or themselves and lose their autonomy in all daily activities. The medical/paramedical professionals (including clinicians, neurologists, and nurses) who participated in the survey were from hospitals and medical units in Zhejiang province, China. The questionnaires were distributed and checked by our trained research assistants to ensure completion.

### Study design

This was a cross-sectional study. The questionnaire was developed based on similar survey studies in China, and the items were generated after a literature review of published surveys (22). The questionnaire (22) was originally translated and culturally adapted from the English questionnaire used in previous research (9). A panel of experts in the field of DoC reviewed and assessed the designed questionnaire. The finalized questionnaire included three sections: participants’ demographics (age, gender, religion, monthly income, level of education, and profession), their perspectives on WLST and AD for UWS patients, and the identification of potential considerations that may influence WLST.

To investigate the perspectives on WLST in UWS patients, we employed the following questions with possible bivariate choices of agreement/disagreement:

1. Is it acceptable to withdraw ANH in patients with UWS (>1 year)?
2. In the event of infection, is it acceptable to withdraw antibiotics in patients with UWS (>1 year)?
3. In the event of cardiopulmonary arrest/cardiogenic shock or other life-threatening situations, is it acceptable not to perform cardiopulmonary resuscitation in patients with UWS (>1 year)?
4. For multiple-option question “What considerations led you to consider WLST?,” a list of choices was given, including patients’ ADs, family’s opinion, financial burden, medical advice, nurse’s advice, the prognosis of disease, duration of DoC, etiology, age of patients, poor quality of life, loss of autonomy, patient’s pain, medical resource, legal feasibility, other people’s view.

The final section of the study focused on professionals’ understanding of ADs. The following questions were asked, and bivariate yes/no choices were given:

5. Have you heard of ADs before?
6. Do you support ADs now after attending the current conference?

### Statistical analyses

Descriptive analyses were conducted to present the demographic characteristics of the participants. Utilizing Cronbach’s alpha, the 11 items’ internal consistency was evaluated. The survey showed good internal consistency, with a Cronbach’s alpha of 0.77. Chi-square tests were used to assess differences within and between categorical variables. The monthly income was divided by a threshold of 5,000 CNY, which is determined based on the disposable income *per capita* published by the government. Binary logistic regression (enter method) was used to examine the associations of agreement with the questions on WLST and ADs with potential predictors such as age, gender, monthly income, region, educational level, and participants’ medical profession. All analyses were performed using SPSS 25.0. The significance level was set at 0.05 (two-sided).

## Result

### Participants

Demographics of the 230 healthcare providers were described in Table 1. 69.7% of our participants were female. Despite a wide diversity of professions, the majority of the participants (93.9%) did not have any religion. Among the recruited participants, 87.8% had bachelor’s degrees or above. In terms of profession, 14.3% were neurologists, 24.3% were clinicians, and 61.3% were paramedical professionals.

**Table 1:** Demographics of the study participants (*N* = 230).

Characteristics	*N* (%)
**Age (year)**	
18–30	220 (95.2%)
31–50	7 (3.1%)
≥50+	3 (1.3%)
**Gender**	
Female	161 (69.7%)
Male	69 (29.9%)
**Monthly income (CNY)**	
<5,000	208 (90.4%)
≥5,000	22 (9.6%)
**Religion**	
Yes	14 (6.5%)
Buddhism	10 (4.3%)
Christianity	3 (1.3%)
Islamic	1 (0.4%)
No	216 (93.9%)
**Education level**	
Senior high school and below	28 (12.2%)
Bachelor degree and above	202 (87.8%)
**Medical profession**	
Neurologist	33 (14.3%)
Non-neurologist	197 (85.7%)
Clinician	56 (24.3%)
Paramedical professional^*^	141 (61.3%)

^*^Including nurse, and physician of other medical disciplines.

### Perspective on WLST

**Figure 1:**
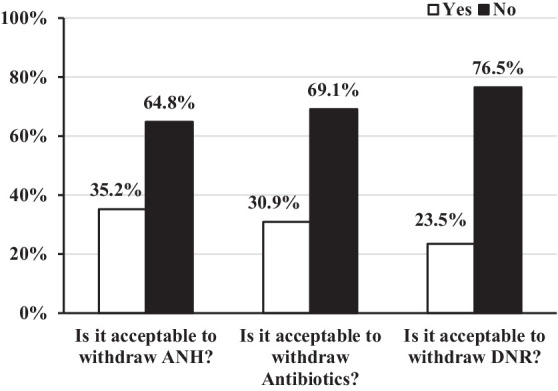
Participants’ attitudes toward withdrawal of life-sustaining treatment in UWS (*N* = 230). UWS, unresponsive wakefulness syndrome; ANH, artificial nutrition and hydration; DNR, do not resuscitation.

A summary of the participants’ attitudes on WLST for UWS patients was presented in Figure 1. 35.2% of the participants reported a positive response about withdrawing ANH. When an infection occurred, 30.9% of participants considered it acceptable to withdraw antibiotics. 23.5% of participants believed that it was acceptable to not resuscitate in the event of cardiac arrest or other life-threatening situations. Withdrawing ANH was preferred over DNR (OR = 1.77, 95% CI: 1.18–2.66, *p* = 0.006). According to chi-square tests, being religious (χ^2^ = 4.837, *p* = 0.016) and having a monthly income of more than 5,000 CNY (χ^2^ = 4.114, *p* = 0.043) were both associated with agreement with DNR order (Table 2). Contrary to what expected, according to the binary logistic regressions, religion was found to be significantly correlated to agreement with DNR order (AOR = 0.31, 95% CI: 0.10–0.97, *p* = 0.044) in culture of China (Table 3). Other factors such as age, gender, education level, and profession did not significantly influence the decision to implement a DNR order for UWS patients. Moreover, no statistically significant differences were observed in the decision to withdraw ANH and antibiotics in UWS patients.

**Table 2:** Participants’ DNR perspectives toward UWS and AD by demographics (*N* = 230).

Item		In the event of cardiopulmonary arrest/cardiogenic shock or other life-threatening, is it acceptable to DNR?			Do you support ADs now after attending the current conference?		
		Yes	No	*p*-value	Yes	No	*p-*value
Age	<30	50 (22.7%)	170 (77.3%)	0.429	211 (95.9%)	9 (4.1%)	0.400
	30–49	3 (42.9%)	4 (57.1%)		6 (85.7%)	1 (14.3%)	
	>50	1 (33.3%)	2 (66.7%)		3 (100.0%)	0 (0.0%)	
Gender	Male	16 (23.2%)	53 (76.8%)	0.946	63 (91.3%)	6 (8.7%)	**0.034**
	Female	38 (23.6%)	123 (76.4%)		157 (97.5%)	4 (2.5%)	
Income	<5,000	45 (21.6%)	163 (78.4%)	**0.043**	194 (96.0%)	8 (4.0%)	0.439
	≥5,000	9 (40.9%)	13 (59.1%)		26 (92.9%)	2 (7.1%)	
Religion	None	47 (21.8%)	169 (78.2%)	**0.016**	208 (96.3%)	8 (3.7%)	0.060
	Yes	7 (50.0%)	7 (50.0%)		12 (85.7%)	2 (14.3%)	
Education	Senior high school and below	6 (24.1%)	22 (78.6%)	0.785	26 (92.9%)	2 (7.1%)	0.698
	Bachelor degree and above	48 (23.8%)	154 (76.2%)		177 (96.2%)	7 (3.8%)	
Profession	Non-neurologist	42 (21.3%)	155 (78.7%)	0.059	191 (97.0%)	6 (3.0%)	**0.018**
	Neurologist	12 (36.4%)	21 (63.6%)		29 (87.9%)	4 (12.1%)	

^*^Chi-square tests. Bold values: there are statistical differences.

**Table 3:** Agreement with the items of DNR and AD concerning UWS by demographics (*N* = 230).

Items	In the event of cardiopulmonary arrest/cardiogenic shock or other life-threatening, is it acceptable to DNR?		Do you support ADs now after attending the current conference?	
	AOR with 95% CI	*P*-value^*^	AOR with 95% CI	*P*-value^*^
Male	0.89 (0.45–1.78)	0.748	0.28 (0.07–1.06)	0.060
Income < 5,000	0.47 (0.18–1.20)	0.114	1.74 (0.31–9.88)	0.533
Religion	3.18 (1.03–9.83)	**0.044**	0.21 (0.03–1.22)	0.081
Non-neurologist	0.47 (0.21–1.05)	0.064	4.45 (1.13–17.59)	**0.033**

AOR, adjust odds ratio; 95% CI, 95% confidence interval. Reference group: Female, income ≥ 5,000, non-religion, and neurologist; predict variables, The answer is “yes.” ^*^Binary logistic regression (enter method). Bold values: there are statistical differences.

For multiple-option question, among factors to consider when WLST, 85.7% of participants reported patient’s AD as the main reason for WLST. 67.8% of participants reported family opinions as the dominant factor for WLST. In addition, 66.5% of participants indicated that financial burdens of continued LST to the family as another main reasons when deciding on WLST. Medical advice, prognosis, patient’s pain, and so on were considered to influence WLST slightly (Table 4).

**Table 4:** Healthcare providers’ considerations regarding withdrawal of life-sustaining treatment in UWS patients (*N* = 230).

Considerations	*N* (%)
Advanced directive	197 (85.7%)
Family wish	156 (67.8%)
Financial burden	153 (66.5%)
Prognosis of disease	103 (44.8%)
Medical advice	102 (44.3%)
Patients’ pain	99 (43.0%)
Poor quality of life	78 (33.9%)
Loss of autonomy	86 (37.4%)
Age of patients	72 (31.3%)
Length of time in DoC	74 (32.2%)
Legal feasibility	74 (32.2%)
Cause of brain damage	71 (30.9%)
Medical resource	55 (23.9%)
Nurses’ advice	44 (19.1%)
Other people’s view	24 (10.4%)

### Perspective on AD

**Figure 2:**
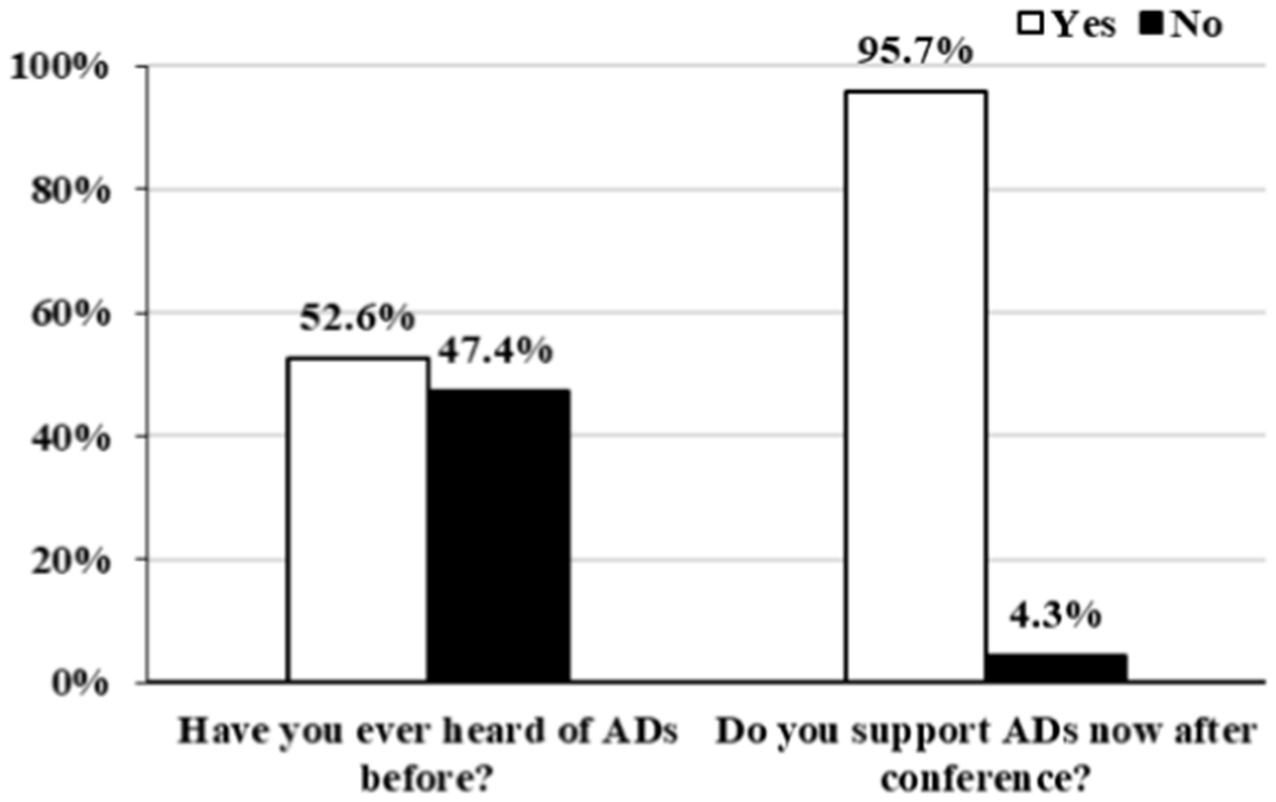
Participants’ attitudes toward advanced directives in UWS (*N* = 230). AD, advanced directives.

47.4% of the participants had never heard of ADs before the conference. Once the participants learned about ADs, nearly all of them (95.7%) agreed that when making medical decisions for patients with UWS, the patient’s AD should be taken into consideration (Figure 2). Compared to males and neurologists, female participants and non-neurologists were more likely to support ADs when considering end-of-life care for UWS patients. Binary regression analysis partly confirmed these results, showing that non-neurologists were more in favor of using ADs compared with neurologists (AOR = 4.45, 95% CI: 1.13–17.59, *p* < 0.05; Table 3).

## Discussion

Although its early introduction in the late 1980s, palliative care in China is still in its infancy and has gotten little attention in recent decades (23, 24). The current study is the first to examine the attitudes of healthcare providers toward end-of-life care among UWS patients in China. According to our analyses, only a small number of Chinese medical/paramedical professionals accepted WLST. Because WLST inevitably leads to death, it may not be in line with the norms of Chinese culture. The findings of this study have important significance for advancing palliative treatment for patients with UWS and for understanding the perspectives of Chinese medical professionals on WLST and ADs.

Less than one-third of our participants (35.2% agreed with withdrawing ANH, 30.9% agreed with withdrawing antibiotics, and 23.5% agreed with DNR orders) reported positive attitudes toward WLST from UWS patients, which notably contrasts with the tendency observed in Europe, where two-thirds of medical and paramedical professionals reported that WLST is acceptable (9). This difference was possibly caused by the significant culture differences (14, 16). A China review concluded that in fact, WLST or refusal to provide LST has been reported to elicit harsh criticism from public opinion (25). Patients’ offspring would be accused of breaking filial piety, clinicians would be charged with murder or duty-related misconduct, and supporters would be branded as anti-humanitarians for failing to save lives and care for the sick (25). Moreover, the misdiagnosis rate in patients with UWS is still high (24.7% according to clinical consensus in UWS in China) (26), even though functional neuroimaging techniques are increasingly providing evidence of residual cognitive processing in these patients (3, 27, 28). A German study revealed that the majority of the clinicians believed that UWS patients could dream, have thoughts and emotions, and sense gustatory and tactile stimuli, including pain (13). The patient’s responsiveness to noxious stimuli might be considered an index of residual cognitive capacity by some and may thus influence attitudes toward WLST, although it might not necessarily be indicative of consciousness. These conflicting reports and diagnostic uncertainties make Chinese healthcare providers discuss WLST with caution.

Comparing our findings to early research on ADs among the general public in China, we observed a significant advancement in the understanding of ADs (29–31). In Anhui province of China (29), 88.61% of participants said they had never heard of ADs in 2020, and in Wuhan city of China in 2012 (31) and 2019 (30), 95.3 and 81.8% of participants, respectively, stated that they had never heard of ADs. On the contrary, in our study only 47.4% of participants had never heard of AD. This may be partially explained by some historical/legal considerations: after the passage of the Shenzhen AD law, several media outlets broke the news of the law’s passage, pushing the article “Shenzhen legislation respects patients’ right to make end-of-life decisions” to the top of search results, with a total of 120 million readers (18). This greatly helped to popularize and promote the concept of AD (18). Moreover, our participants were healthcare providers rather than the general public as in studies of Wuhan and Anhui which may have contributed to the high rate of awareness on AD (29–31); Indeed, in recent years, China has been increasing training of health professionals on palliative care (32). Finally, our participants were much younger compared to participants in the studies in Wuhan (being 65 years of age or older) or in the study in Anhui (29–31). A potential explanation might be that young individuals have more access through social media and the web in general to new information.

After our conference, 95.7% of the participants agreed that ADs should be taken into account when clinicians or families are making medical decisions for patients with UWS. Despite a study of the city of Wuhan in which only 22.4% of cancer patients approved the concept of ADs, 80.2% of participants from Hong Kong and 73.6% of participants from Macau claimed that they were willing to sign an AD (21, 31, 33). The reasons why the findings are drastically different from ours could be related to the differences in patient’s illnesses. For example, cancer patients are conscious and able to express their wishes, in contrast to UWS patients who, suffering from several cognitive/physical impairments, can rarely express their preferences after the injury. Indeed, LST is frequently started within months to years from the injury when the patients might have already lost the capacity to make medical decisions, which often translates in disregarding patient’s rights, autonomy, and dignity (34, 35). Family members and other sources of medical information might not be able to accurately predict patients’ personal preferences in terms of LST/WLST (36). ADs can therefore provide reliable support for clinicians and patients’ families when making end-of-life decisions. However, because brain injuries typically occur unexpectedly in young and healthy individuals, rarely have DoC patients been able to make an early declaration of AD (37). Therefore, advocacy, legislation, and popularization of AD-related procedures are essential to the work ahead.

Differences in findings regarding the support of AD between the current study and previous research [support for ADs was found to be higher here than in Wang et al. (29) and Ni et al. (30, 31)] might also be explained by differences in adopted methodologies. Previous studies, in fact, have invariably used a short, written definition to introduce participants to AD. Here, we explained at length, during an oral presentation held in person, the concept of AD. Hence, the discrepancy between ours and previous findings may be explained by inadequate/inefficient education about AD. Our results showed that a brief introduction to ADs is very effective in improving health professionals’ positive attitudes toward WLST and ADs in general. We therefore recommend introducing the concept of AD in detail to the participants prior to administering the survey, as done in this study, to ensure that participants gain an accurate understanding of the concepts used, yielding more valid results. With regards to WLST, our study found that healthcare providers prefer to withdraw ANH than DNR. This is consistent with the findings of a recent cross-national survey in Asia, in which clinicians in China were more inclined to withdraw or withhold ANH but less likely to limit the use of active treatments such as cardiopulmonary resuscitation, dialysis, and vasopressors (5). This preference to withdraw ANH compared to DNR might be explained by the different impact on the patient. As the termination of ANH does not result in immediate death, it might help overcome the taboo of talking about death that is inherent in Chinese culture (38).

In contrast to what one may expect, in our study, non-religious participants were against WLST. While most Chinese people may not have any religious beliefs, Confucianism has deeply influenced Chinese culture, philosophy, social values, and ethical considerations, resulting in an ethical system family-based and harmony-oriented, with strong emphasis on filial piety (5, 14, 16). According to Confucianism, the interests of family members are closely connected, and death can lead to family disruption. Similarly, we also document that significantly more non-neurologists preferred to support patients’ AD in WLST as compared to neurologists. A possible explanation might reside in the additional legal responsibilities that neurologists have when making end-of-life decisions.

An interesting finding is that economic factors appear to play a significant role in the end-of-life decision-making process. For example, most participants indicated financial burdens as a main reason when deciding on WLST, and participants whose monthly income is less than 5,000 CNY are more reluctant to withdraw treatment compared to those with higher incomes. Previous studies from Asian (including China, Japan, Korea, Indian and so on) have also shown that physicians in low-and middle-income countries and regions are more likely to vacillate between continuing life-sustaining treatments for terminally ill patients at the end of life and withholding or withdrawing them in otherwise salvageable patients because of the lack of resources (39). The charity-oriented opinion in China leads most families to lean toward providing long-term life-sustaining treatment for patients. Despite the partial reimbursement (60%–80%) from the healthcare system, the long-term expenses still place heavy economic burdens on families. Therefore, healthcare providers have to consider finical burden as an important factor and provide decision-making suggestions to family based on the patient’s prognosis, in order to help family surrogates make end-of-life decisions.

### Strengths and limitation

We conducted a pioneering survey on the attitudes of Chinese healthcare providers toward end-of-life care for UWS patients. Given the paucity of studies conducted on the end-of-life decisions in UWS patients, this study substantially contributes to raising the state of knowledge regarding AD and WLST choices of Chinese health providers. Nevertheless, several limitations of the study remain. We only gathered data from healthcare professionals in Zhejiang, China; hence, generalizability may be somewhat constrained, and additional research in additional areas/regions of China is required. Our study relies on predefined questions and answers, potentially do not capture details and nuances, therefore we need to investigate more detailed medical surrounding, social-culture and diagnosis and prognosis knowledge in an open-ended way to design practical assistance for this cohort of individuals. Furthermore, future research with larger sample size is warranted.

## Conclusion

We found that only less than one-thirds percentage of healthcare providers expressed supportive/positive attitudes toward WLST for patients with UWS in China. These results raise significant concerns about the attitudes deeply embedded in clinicians, even though they cannot be interpreted as reflecting current clinical practices or public attitudes. In contrast, after the presentation on ADs at the conference, the attitudes of healthcare providers toward ADs for patients with UWS are generally positive.

## Data availability statement

The raw data supporting the conclusions of this article will be made available by the authors, without undue reservation.

## Ethics statement

This study was approved by the Ethics Committee of Hangzhou Normal University (Ref No. 2022050). Informed consents were obtained from the participating healthcare providers. Completion of the questionnaire was anonymous. The study was conducted strictly according to the World Medical Association’s Declaration of Helsinki.

## Author contributions

ML: Writing - review & editing, Writing - original draft. SD: Writing - review & editing, Writing - original draft. LW: Writing - review & editing, Investigation, Conceptualization, Methodology, Data curation. HD: Writing - review & editing, Data curation, Supervision.
