# Supplementary material for: Perspectives of healthcare providers on withdrawal of life-sustaining treatment and advanced directives for unresponsive wakefulness syndrome in China
Source: Front Neurol. 2024 Aug 14;15:1358747. doi: 10.3389/fneur.2024.1358747 (PMC11350639; doi:10.3389/fneur.2024.1358747)
Supplement: Supplementary file 1 [file Data_Sheet_1.PDF]

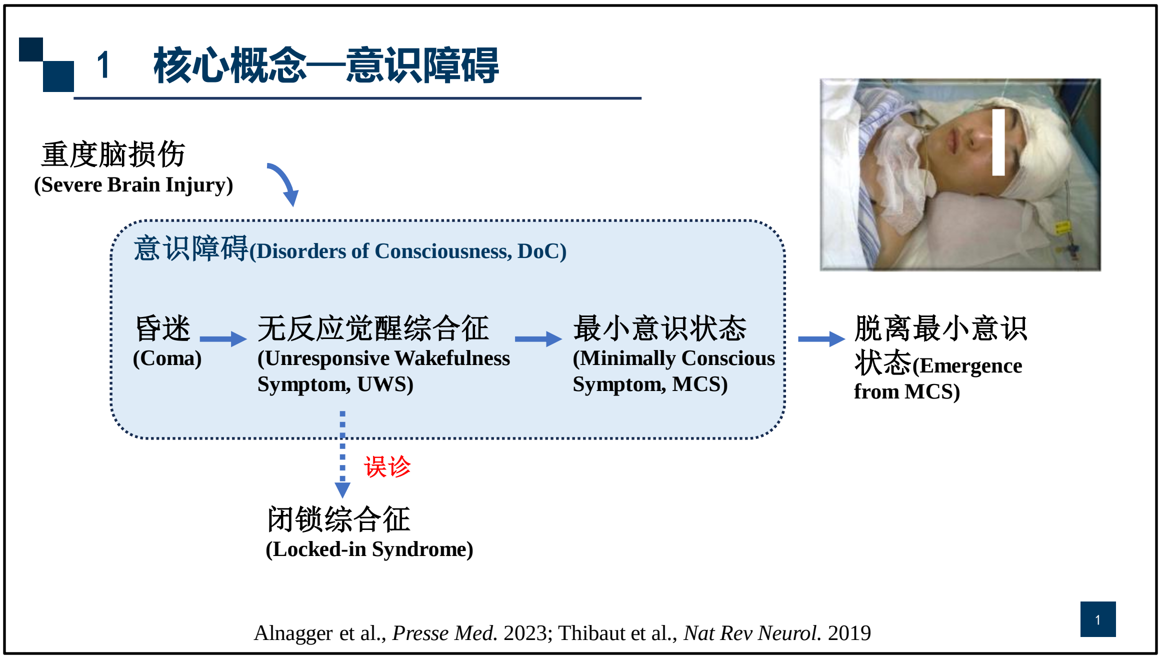

With the advancement of emergency medical technology, a significant number of patients with severe brain injuries survive and develop disorders of consciousness. After passing through a brief coma period, these patients open their eyes but sometimes remain completely unaware of their surroundings and unable to respond to any commands. This condition is referred to as Unresponsive Wakefulness Syndrome (UWS), commonly known as a vegetative state. When patients begin to show inconsistent responses to commands, they are considered to be in a Minimally Conscious State (MCS), indicating some behavioral evidence of consciousness, though their responses are typically unstable. Once patients are able to provide consistent and correct responses, they are said to have emerged from the minimally conscious state (EMCS) and are no longer considered to have a disorders of consciousness. From left to right, there is a gradual improvement in cognitive and motor functions.

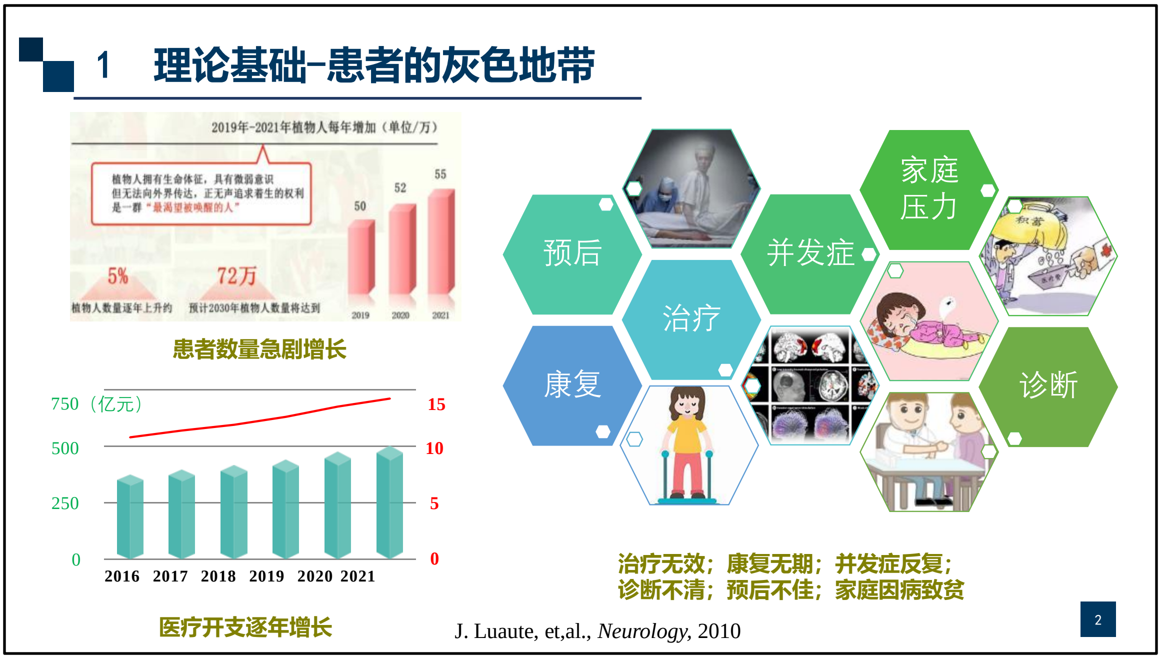

After a large number of patients survive, the demand for medical care increases sharply. Due to unclear pathological mechanisms, diagnosis and treatment are extremely challenging, with up to 40% of behavioral diagnoses not matching the doctors' diagnoses. Aside from the secondary evidence supporting the effectiveness of Amantadine and Zolpidem, there is no rigorous evidence-based support for other treatments. Long-term bed rest causes all patients to experience complications, with one-third of patients dying from these complications within two years. Patients often endure a decline in quality of life and loss of dignity throughout their entire survival period. Families also face significant caregiving and financial burdens.

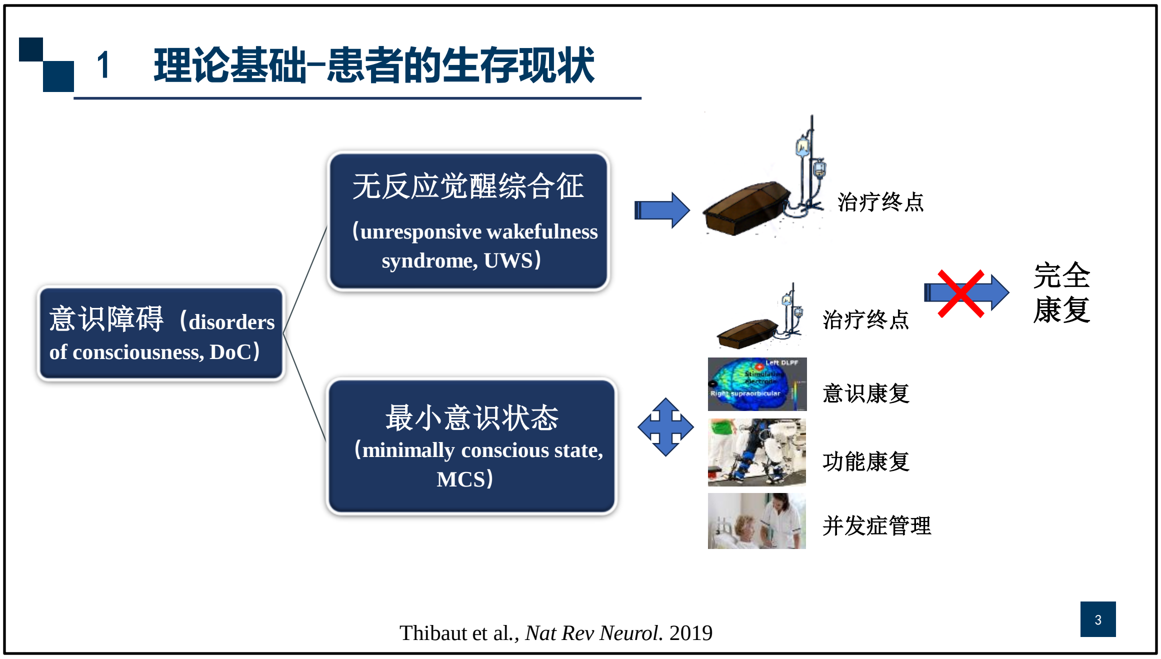

In cases where the diagnosis is unclear, treatments are ineffective, and complications recur, most patients with Unresponsive Wakefulness Syndrome eventually succumb to death. Even though patients in a Minimally Conscious State have a slightly better prognosis, they may remain in this state for decades, potentially deteriorating or dying from complications in the short term. Almost none of the patients achieve complete recovery; however, with life-sustaining treatment, they can survive for many years.

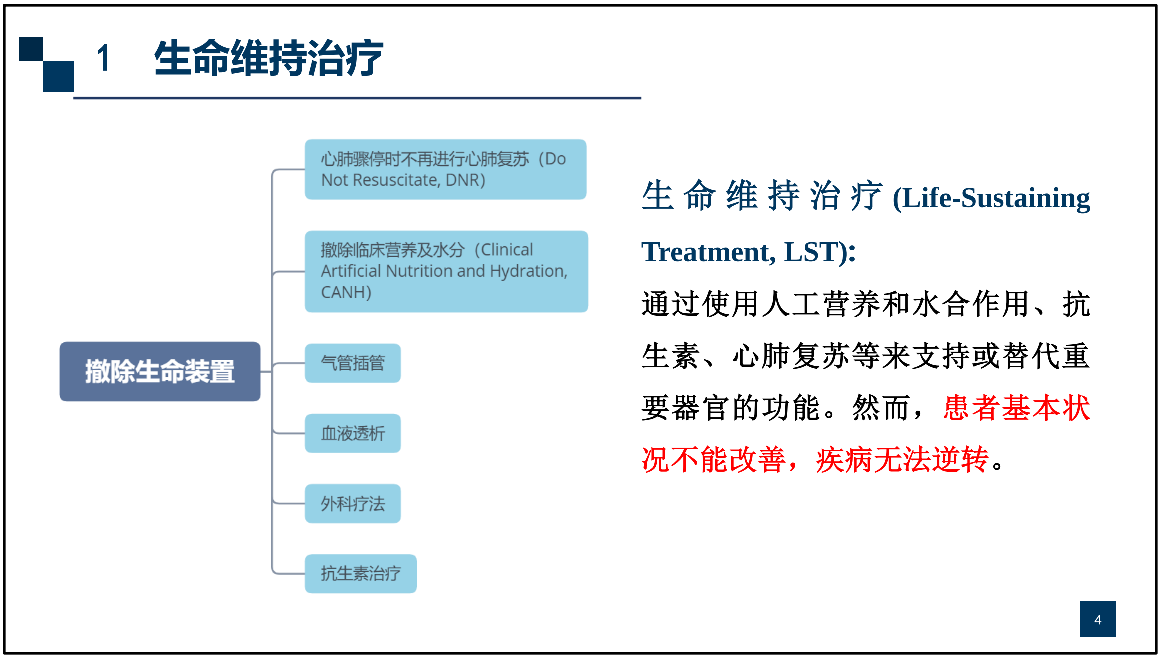

Life-sustaining treatment essentially involves using artificial means to replace the functions of vital organs, while the underlying condition of the disease cannot be reversed. Therefore, relying on life-sustaining treatment to keep patients alive for long periods in a state of complete loss of autonomy has sparked intense ethical, social, and medical debates.

1 患者的临终问题

台灣「安樂死立法」的漫漫長路，從「王曉民的悲劇」說起

發現自己可能比女兒早走，父親轉而接受「安樂死」

1982 年 8 月 11 日星期三，在西仕颱風為臺灣北部帶來的大雨之中，**趙錫念在全家歷經 19 年生理和心理的巨大煎熬，加上自己罹患心臟病並輕微中風兩次之後，發覺自己有可能會比女兒先走一步，因而從原本不願意對王曉民進行安樂死，轉念希望女兒能夠好好地離開人世，所以便到臺北的中國人權協會提出申請，希望協會可以協助讓她的女兒進行安樂死。**

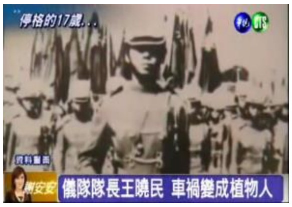

17岁（1963年）车祸成植物人

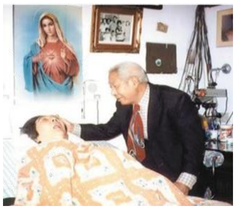

34年后，80岁的父亲离世

雖然數年前趙錫念的請願沒有成功，但立法院在 1986 年 5 月 12 日首次舉辦了「安樂死應否立法問題」座談會，邀請各界專家就安樂死立法進行研究，並印製成專冊作為立法參考。到了 11 月，在趙錫念第一次向立法院請願失敗之後，趙錫念仍不死心，繼續請願，同時，這 3 年間也有其他社會人士因為同情王曉民或自身疾病的緣故，而向立法院請求安樂死立法。

到了 11 月 28 日，總共有 7 個安樂死請願案終於被排入議程，在經過激烈的辯論後，全案決定留待下次會議繼續討論。但這個下次，一等就是 10 年。

善終，可能嗎？

來不及親眼見證安樂死立法成功，趙錫念在 1996 年 6 月因為癌症去世，享壽 74 歲。3 年後，81 歲的王雷雲也因為呼吸衰竭而過世。**由於 30 多年來日夜照顧王曉民的經驗，兩人在去世之前都拒絕急救。王曉民則由 3 位妹妹與看護繼續無微不至地照顧，直到 2010 年 3 月，在病榻躺了 47 年後，於 64 歲離世。**

由於趙錫念多年來努力推動安樂死，臺灣社會開始思考安樂死的可行性。不過，王曉民的狀況其實相當特殊。因為植物人沒有行為的能力，所以其實並不是安樂死的對象，從當時的報導和後來舉行的安樂死相關研討會，大多把王曉民的例子作為照護體系對植物人的照顧議題，但這樣其實並沒有考量到植物人的生活品質。

躺了半个世纪的“植物人”--台湾王晓民

5

For example, in Taiwan of China, a patient named Wang Xiaomin, who was in a vegetative state, survived for 47 years relying on life-sustaining treatment but never regained consciousness. His family's half-century wait ended in vain, and the prolonged caregiving eventually devastated the family. This case represents the tragic reality faced by thousands of families in China dealing with disorders of consciousness. However, this may not be what the patient desires.

1 患者的临终问题—生前预嘱

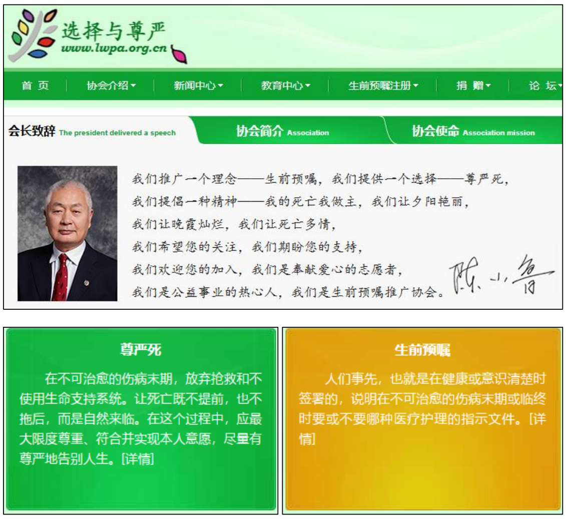

生前预嘱(Advanced directives)：
人们事先，也就是在健康或意识清楚时签署的，说明在不可治愈的伤病末期或临终时要或不要哪种医疗护理的指示文件。

尊严死(Dignified death)：
在不可治愈的伤病末期，放弃抢救和不使用生命支持系统。让死亡既不提前，也不拖后，而是自然来临。在这个过程中，应最大限度尊重、符合并实现本人意愿，尽量有尊严地告别人生。

6

The right to die with dignity has always been the focus of domestic efforts. Dignified death means giving up rescue efforts and not using life support systems in the terminal stage of incurable illness or injury, allowing death to occur naturally without hastening or delaying it. In this process, the individual's wishes should be respected, adhered to, and realized to the greatest extent possible, allowing for a dignified farewell to life. Previously, Shenzhen passed a law on advance directives to protect patients' rights. Advance Directives refer to documents signed by individuals in advance, typically when they are healthy or of sound mind, that specify their preferences for medical care in the event of incurable illness or at the end of life.

1 限制生命维持治疗研究进展

Audience (*n*)	Agreement (%)
Physicians (208)	94
Physicians (1,027)	94
Neurologists (169)/Medical directors (150)	88/89
Internists (326)	80
Neurologists (169)/Medical directors (150)	10/13
Physicians (115)/Nurses (127)	90/89
Physicians (345)	92

欧洲、加拿大、美国等西方国家中，严重脑损伤患者限制治疗的比例高达80%以上。

日本、韩国等亚洲国家也相继开展了此类研究

美国

- **Karen Quinlan**
- **Nancy Cruzan**
- **Terri Schiavo**

欧洲

- 意大利女性患者 **Eluana Englaro**
- 法国男性患者 **Vincent Lambert**

东亚

- 日本是亚洲第一个承认有条件安乐死的国家
- 韩国对“尊严死”进行了相关立法研究

中国

- 2000年我国台湾地区通过了地方性的《安宁缓和医疗条例》
- 中国香港特别行政区于2000年 1月13 日，通过了《被动安乐死守则》

Demertzi A, et al. *Journal of Neurology*. 2011

In Europe and North America, 70-97% of brain injury patients in intensive care units die following the withdrawal of life-sustaining treatment. Previous studies in these regions indicate that 80% of healthcare workers believe it is appropriate to limit life-sustaining treatment for patients with disorders of consciousness. Cases like those of Quinlan and Schiavo in the United States have played significant roles in shaping legislation in these countries. Similar studies have been conducted in Japan and South Korea, and in 2000, Taiwan and Hong Kong introduced relevant local laws.

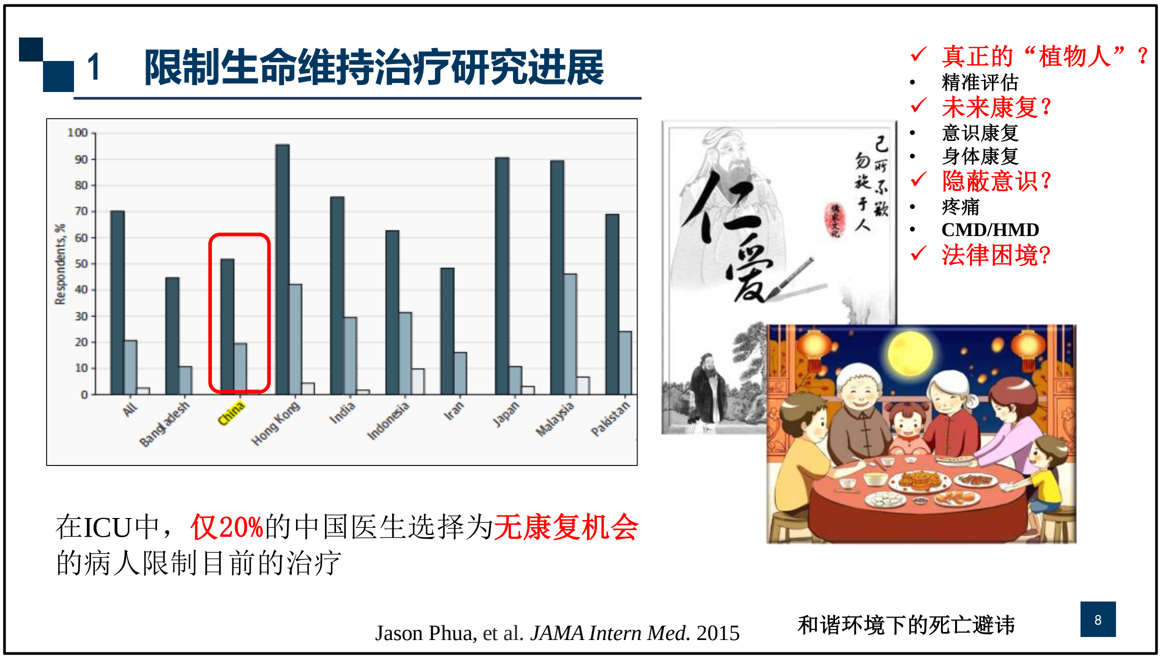

In mainland China, research on limiting treatment is relatively sparse. The figure shows that only 20% of doctors in China believe limiting treatment is feasible, based on a study of critically ill patients in East Asia. Due to China's harmonious ethical views and the numerous unresolved issues surrounding disorders of consciousness, there may be significant differences in perspectives between China and Western countries on limiting treatment for patients with consciousness disorders.
